# Construction of an experimental set up to perform full body micro cannulations of sub-millimetric vessels in an anatomical research setting

**DOI:** 10.1371/journal.pone.0358264

**Published:** 2026-09-11

**Authors:** Michael E. J. Stouthandel, Danial Forouhar, Charlotte Debbaut, Jurgen Deviche, Liesl De Graeve, Bernard Depypere, Maarten Meire, Dominique Adriaens, Tom Van Hoof

**Affiliations:** 1 Department of Human Structure and Repair, Ghent University, Ghent, Belgium; 2 Cancer Research Institute Ghent (CRIG), Ghent University, Ghent, Belgium; 3 Evolutionairy Morphology of Vertebrates, Ghent University, Ghent, Belgium; 4 IBiTech-bioMMeda, Ghent University, Ghent, Belgium; 5 Department of Plastic Surgery, Ghent University Hospital, Ghent, Belgium; 6 Breast Clinic, Ghent University Hospital, Ghent, Belgium; 7 Department of Oral Health Sciences, Ghent University, Ghent, Belgium; University of Missouri, UNITED STATES OF AMERICA

## Abstract

**Introduction:**

A better 3D visualisation of the lymphatic system could provide more accurate cancer treatment approaches, but this requires contrast agent injections. Since lymphatic vessels are very small, cannulations to administer contrast agent are very difficult to perform manually. As such, a dedicated set up for lymphatic vessel micro cannulations is required.

**Materials and methods:**

A micromanipulator rig for full body micro cannulations was constructed. The rig consists of an aluminium frame that can be positioned over a dissection table. It combines the micromanipulator with a sliding gantry plate and a double jointed positioning system to provide as many possible angles of approach as possible. Fluid pressure control devices and a surgical microscope can also be included in the set up.

**Results:**

Micro cannulations on a full body could successfully be performed using the micromanipulator rig. After only a short learning curve to explore the movement possibilities/range of the device, the micromanipulator rig quickly outperformed manual micro cannulations.

**Conclusions:**

Given the short learning curve and the success rate of the micro cannulations with the micromanipulator rig, we would strongly recommend other microvascular researchers to use the micromanipulator rig for future experiments involving micro cannulations.

## Introduction

Lymphatic atlases created in the 1700’s and 1800’s were long thought to be flawless, due to their level of detail. As such, they remain the primary source of lymphatic knowledge for both anatomists and clinicians [1]. Original lymphatic anatomical research remains very limited for this reason and these original atlas plates have not been reviewed or updated in centuries [2]. Recent research shows that the lymphatic knowledge contained in these old lymphatic atlases includes known errors [3] or incomplete information [4]. Considering the important role of the lymphatic system in cancer treatment and the major advances in medical imaging technology, a re-evaluation of lymphatic anatomy is urgently needed and feasible. More accurate 3D depictions of lymphatic pathways in relation to the surrounding anatomy could improve treatment accuracy for radiotherapy and surgical oncology. It can also improve anatomy education quality for future healthcare professionals [2,5–7].

Several challenges exist for lymphatic system research. Lymphatic vessel diameters range from 4.8 mm in the thoracic duct [8], to only 20 μm in lymphatic capillaries [9]. Due to their transparent walls and small size, peripheral lymphatics are difficult to identify during a dissection without the help of dye or contrast agent [2,10]. Likewise, uncontrasted lymphatics are invisible on CT scans [11] and they cannot reliably be distinguished from other microscopic vessels on 7T MRI [12], or micro CT [13,14]. Intralymphatic contrast agent injection is therefore essential for accurate 3D mapping of the lymphatic system [5]. Because successful cannulation is technically challenging, most lymphatic flow studies focus on the thoracic duct [6,15–17], while smaller vessels remain poorly investigated [7].

Accessibility poses an additional obstacle. Deep lymphatics, like the subclavian lymphatic trunk (SLT) that drains the axilla and breast and is highly relevant for breast cancer treatment [7,18,19], are difficult to cannulate due to their close proximity to the ribs and clavicle. This accessibility problem is reflected in the literature: The latest dedicated publication on the SLT dates back to the 1990’s and it lacks both digital 3D depictions and high quality photographic documentation [20]. The historical anatomical record is equally affected. Sappey omitted the SLT entirely from his 1874 landmark atlas ‘Anatomie, physiologie, pathologie des vaisseaux lymphatiques’ [21]. Mascagni’s 1787 atlas ‘Vasorum Lymphaticorum Corporis Humani Historia et Ichnographia’ [22] only includes the SLT in limited detail, which is insufficient to prepare for an anatomical SLT dissection, or a delicate oncological procedure.

Microscopic magnification improves vessel visualisation, but highlights a different challenge. Intrinsic hand tremor frequently damages lymphatic vessels during cannulation, leading to failed contrast injections. A micromanipulator can reduce hand tremor, but current micromanipulators are designed for benchtop microscopes. To study the SLT, a full body approach is required, because the target area (neck to axilla) cannot be positioned on a benchtop microscope. As such, there is a need for a dedicated set up combining microscope assisted micromanipulation with enough degrees of freedom to access lymphatic vessels throughout the body, regardless of depth or possible obstructions (ribs/clavicle). Integrating a component to control fluid pressure and microscopic recording possibilities would also enable the study of contrast agent propagation inside the lymphatic vessels. This type of study remains very rare for deep lymphatic vessels like the SLT [16] and it could provide additional insight into lymphatic vessel and valve properties in an in situ, human cadaver setting.

To the best of our knowledge, an experimental set up that combines full body micro cannulation, controlled intralymphatic contrast agent introduction and microscopic recording for anatomical research on human specimens does not yet exist. Therefore, the objective of this research is to create such a set up, using a multidisciplinary approach. This set up will enable controlled contrast agent introduction in poorly described, deep lymphatic vessels. Properly contrasted lymphatic vessels will allow accurate 3D reconstructions of the lymphatics in relation to the surrounding anatomy, based on MRI + (micro)CT imaging in future studies [23]. Improved depiction of lymphatic structures like the SLT can provide a more accurate foundation for radiotherapy and oncologic surgery for (breast) cancer patients.

## Materials and methods

### Experimental set up design

**Fig 1:**
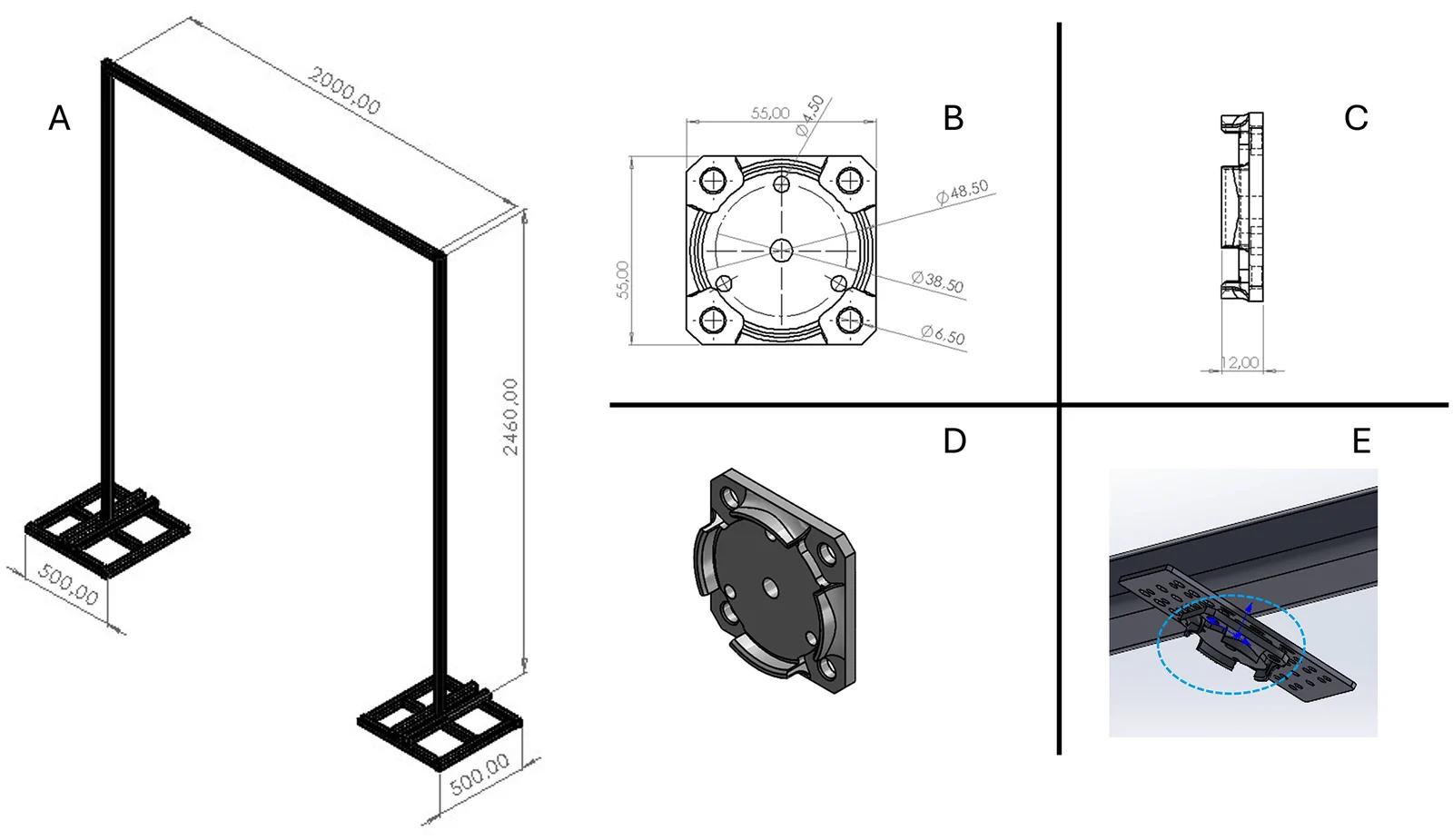
Configuration of extruded aluminium frame, 3D print design for adaptor piece connecting the micromanipulator to the gantry plate and adaptor piece as attached to the gantry plate. Panel A shows the extruded aluminium frame. To build this, the following materials were needed, using 40 x 40 mm extruded aluminium with T/V slots for all of them: 8x 460 mm, to form the outline of the 2 squares at the bottom of the rig. 2x 420 mm for the horizontal bar inside the 2 squares at the base. 4x 500 mm, to stabilize the vertical bars on top of both the square bases (2 bars on each square base). Finally, 2x 2460 mm for the vertical bars and 1x 2000 mm to form the horizontal bar that connects the vertical bars and provides the surface that will be suspended above the dissection table. The extruded aluminium components can be connected using M4 socket cap head bolts, M4 drop in tee nuts and 90 degree cast corners. Panel B shows the top view, panel C shows the side view and panel D shows the 3D rendering of the adaptor piece used to connect the micromanipulator to the gantry plate. The 3 holes within the circle align with the screw configuration used to attach the micromanipulator stand to the magnetic base, while the 4 outer holes align with the hole pattern of the gantry plate. This allows the micromanipulator stand to connect to the gantry plate. Panel E shows the 3D printed adaptor piece (circled in blue) as attached to the gantry plate.

The commercially available components required to construct the full body micro cannulation set up were incompatible without modification. The micromanipulator version M3301R (World Precision Instruments, Friedberg, Germany) was only available on a magnetic stand, intended for use with a benchtop microscope. The magnetic stand needs to rest on the table surface. This severely limits the degrees of freedom of the device when there is also a full body on that same table. Positioning the micromanipulator above the body circumvents this issue, ensuring more degrees of freedom. Because micro cannulation requires high positional stability, as the slightest tremor during the procedure can puncture the vessel, a rigid overhead suspension system was required. Rigid 3D printer frames share the same positional stability requirements, so similar materials were chosen. To ensure maximum stability and compatibility, 40 x 40 mm T/V slot extruded aluminium profiles (Ooznest Limited, Brentwood, United Kingdom) were used in the configuration shown in Fig 1A. Four 10 kg weights (2 per square base) were added to further increase stability.

To integrate the micromanipulator with the suspension rig, a V-slot gantry plate with Delrin wheels (Ooznest Limited, Brentwood, United Kingdom) was mounted on the horizontal bar of the suspension rig. The gantry plate contains a premade hole pattern for attaching attributes. This pattern was used for a 3D print design, in combination with the configuration of the screws connecting the micromanipulator stand to the magnetic base. This 3D print allowed the micromanipulator to attach to the gantry plate, instead of the magnetic base. The design for this 3D printed device is shown in Fig 1B, 1C and 1D. Fig 1E shows how the 3D printed connector is attached to the gantry plate. See ‘3D printing information’ below for additional details regarding the printer specifications and settings.

**Fig 2:**
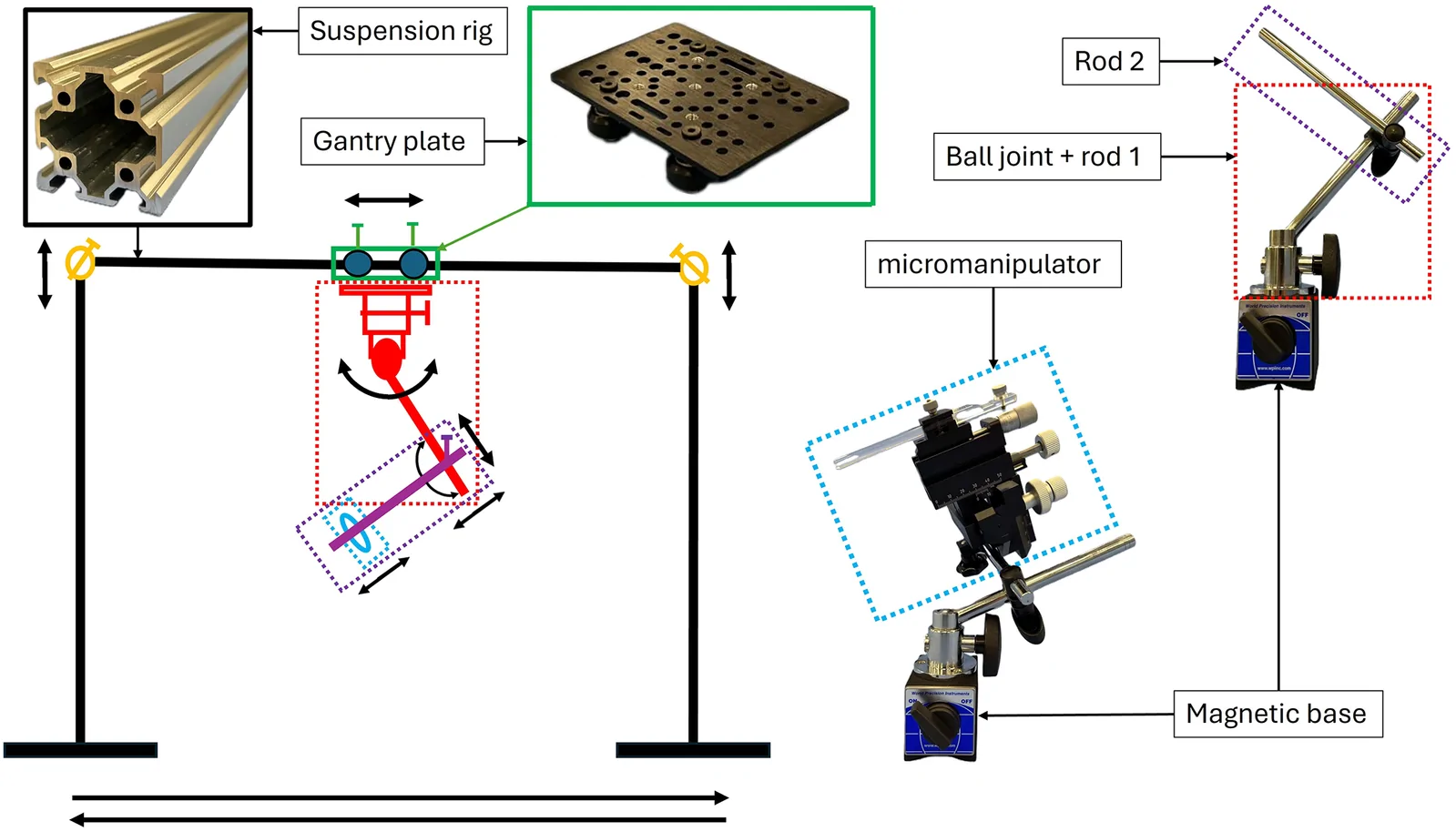
Schematic overview of the micromanipulator rig, highlighting the different components and movement options. The black bars represent the suspension rig, built from 40 mm x 40 mm extruded aluminium profiles (see Fig 1). This frame can be positioned over the dissection table with a full body on it. The horizontal bar can move up and down to accommodate bodies of different sizes. The green outline represent the gantry plate attached to the suspension rig. It can move from left to right on the horizontal bar. The vertical bars can be positioned next to each other, or diagonally, depending on the required angle of approach. The 3D printed connection piece (not shown for clarity) connects the gantry plate to the positioning rods. The positioning rods have been removed from the heavy magnetic base, so they can hang from the gantry plate. The gantry plate can be locked in place by tightening the wheel bolts. The ball joint attached to rod 1 with a diameter of 12 mm (red) provides a full 360 degrees of rotation + tilting options. Rod 2 with a diameter of 10 mm (purple, supplied with micromanipulator) can be attached perpendicularly to rod 1. It can move up and down on rod 1 to further adjust the height. It can also tilt towards, or away from rod 1. Rod 2 can be added, or removed from the set up if required. The micromanipulator was ordered with a 12 mm diameter opening and it can be attached to rod 1, or rod 2. To attach it to rod 2, a 3D printed conversion piece can be used to adapt the size of the opening. When attached to rod 1, the micromanipulator (blue) can make a full 360 degree rotation around the rod. It can also move up and down on the rod to further adjust the height. With this set up, all possible approaches to an area of the full body are theoretically feasible. ‘T’ shapes represent locking mechanisms to lock the associated parts in place.

Fig 2 gives a schematic overview of the complete experimental set up (micromanipulator rig) and the movement options it has while positioned over a dissection table containing a full body. The full experimental set up contains the micromanipulator that is attached to the sliding gantry plate and mounted onto the suspension rig.

### Cannulation equipment compatibility

**Fig 3:**
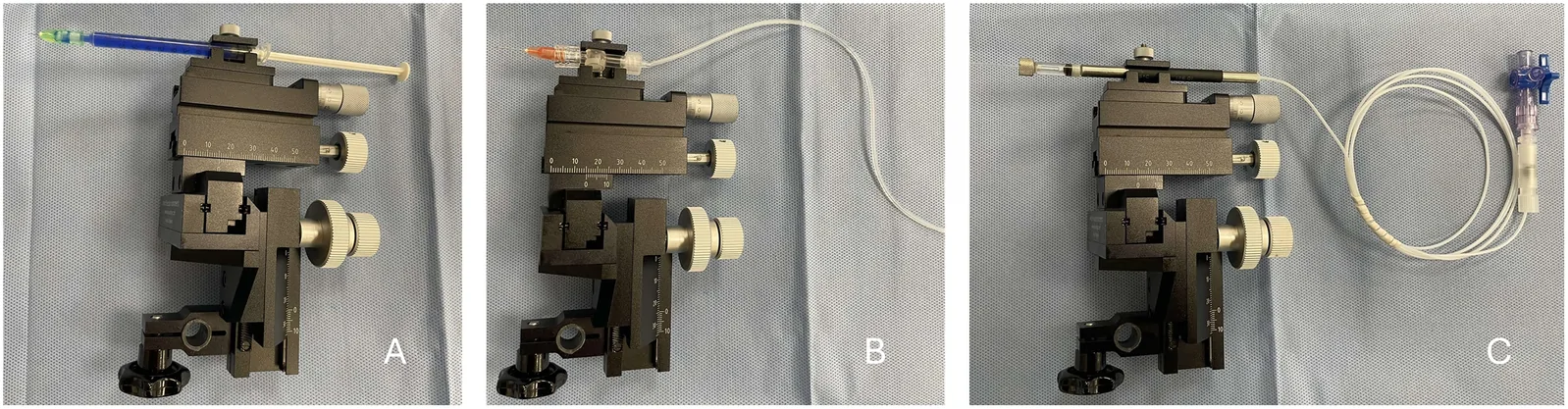
Different configurations of cannulation equipment attached to the micromanipulator, to allow a wide range of lymphatic experimental options. Panel A shows a contrast filled syringe + needle attached to the micromanipulator. This configuration can be used for experiments where the injection pressure is not relevant. Panel B shows a needle connected to tubing attached to the micromanipulator. This configuration can be used in lymphatic experiments where the injection pressure needs to be regulated. Needle size in A and B can be adapted to the size of the vessel to be cannulated up to a diameter of 33G. Panel C shows a glass capillary, connected to the MMP kit + tubing, attached to the micromanipulator. This configuration can be used when very small lymphatics need to be cannulated (smaller diameters than the commercially available needles). The MMP kit configuration can also be used in combination with pressure control devices.

Different lymphatic experiments require different cannulation equipment, so the micromanipulator set up needs to be adaptable to accommodate these different types of experiments (Fig 3). A needle + syringe filled with contrast agent (Fig 3A), can be used if the fluid pressure is not relevant to the experiment. The needle size can be adapted to the size of the vessel. When fluid pressure needs to be regulated, Luer lock compatible coupling pieces and tubing can connect the needle to an external pump, or fluid column (Fig 3B). For an example of a fluid pressure control device used for lymphatic research, the reader can refer to [7,16]. The smallest commercially available needle (33G) is too large to cannulate the smallest lymphatic vessels. To cannulate the smallest lymphatic vessels, pulled glass capillaries, as described by Suami et al [24], can be mounted on the micromanipulator using an MMP kit coupling piece (World Precision Instruments, Friedberg, Germany) (Fig 3C).

**Fig 4:**
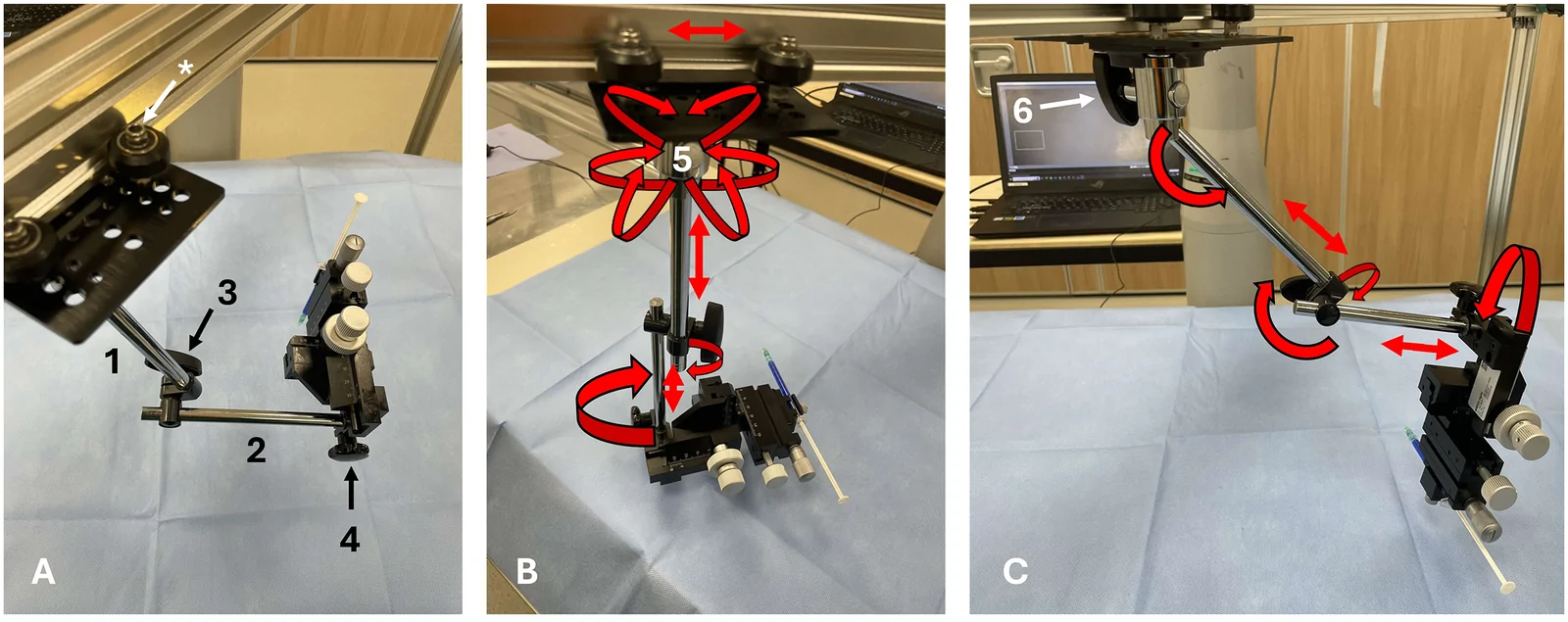
Dry run with the micromanipulator on the suspension rig to test the movement capabilities. Panel A shows an overview of the set up, hanging from the suspension rig, panel B and C show the movement capabilities of the different parts. The ball joint [5] provides 360 degrees of motion to rod 1 in multiple directions (represented by the 6 red arrows) and it can be locked in place using dial 6. In panel B it is shown in neutral position, pointing downwards. The gantry plate can move from left to right on the horizontal bar and it can be locked in place by tightening the individual wheel bolts (*). The connection between rod 1 and 2 that contains dial 3 to lock it in place, enables rod 2 to rotate around rod 1 in 360 degrees and it can also make rod 2 slide up or down on rod 1. The micromanipulator can rotate 360 degrees around rod 2 and it can move up and down on rod 2 if it is not locked in place by dial 4. Panel C shows that the ball joint can also tilt within the 360 degree motion range. Connection piece 3 also allows tilting up and down between rod 1 and rod 2, as shown in panel C. Finally, the micromanipulator can be attached to rod 2 in 2 different ways, to enable an approach from above (panel A), or from below (panel C). The 3 silver coloured dials on the micromanipulator are used for the final, precise X, Y and Z-axis movements required for cannulating small vessels. By using the 2 connection rods, lateral/sideways approaches are also possible as shown in panel B. 1 = rod 1 connected to the ball joint, 2 = rod 2, 3 = dial to lock rod 2 in place on rod 1, 4 = dial to lock micromanipulator in place. 5 = ball joint, providing the most degrees of freedom, 6 = dial to lock the ball joint in place. * = locking mechanism/tightening bolt for a wheel of the gantry plate.

After integrating the suspension rig, the micromanipulator and the cannulation components (micromanipulator rig), a dry run was performed without a body to evaluate the ranges of motion that were practically feasible. Fig 4 shows the different ranges of motion of the micromanipulator rig during this dry run, with additional illustrations of the movement options.

### Surgical microscope, photo/video and livestream option integration

**Fig 5:**
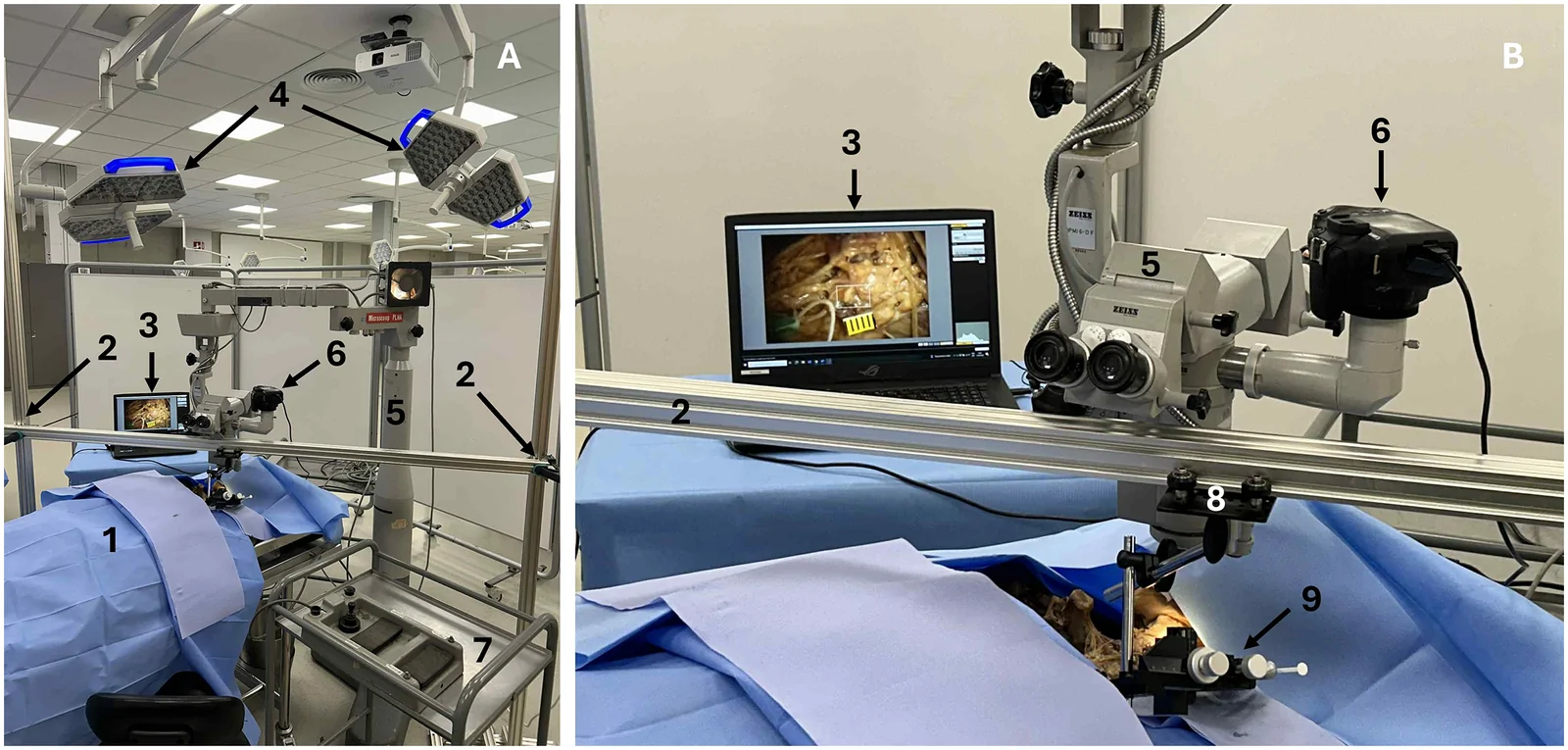
Full micro cannulation set up, with integrated surgical microscope and camera connection for photo and video recordings of experiments and a live view of the dissection field. The full set up is placed around/over a dissection table with a full body and the micromanipulator is positioned to perform a lymphatic cannulation. Panel A shows a full overview of the set up and panel B shows a close up of the part with the micromanipulator and eyepieces of the microscope, to give a better idea of what the researcher works with during the experiment. 1 = dissection table with a full body, positioned under the micromanipulator rig, 2 = micromanipulator rig, 3 = laptop with live feed of the microscope view, 4 = additional light sources in case the light from the microscope isn’t sufficient, 5 = surgical microscope, 6 = camera attached to the microscope using an APS-C adaptor, 7 = control panel for the surgical microscope to enable zooming and focusing the view, 8 = gantry plate, 9 = micromanipulator in position for a lymphatic cannulation.

An OPMI 6-DF surgical microscope (Zeiss, Oberkochen, Germany) was incorporated to prepare for full body experiments. The 5x – 12x magnification of the field of view was required to guide accurate cannulation of sub-millimetric vessels, which cannot be achieved with the naked eye. For image/video acquisition, a Canon EOS 2000D professional camera (Canon, Tokyo, Japan) was attached to the surgical microscope, using an APS-C adaptor (Zeiss, Oberkochen, Germany). The surgical microscope is not connected to the micromanipulator rig and it needs to be positioned so that it can bring the target vessel into clear focus. This means that the bottom part of the microscope cannot touch the horizontal bar, or the gantry plate, as this will obstruct the focusing and zooming operations needed to get the target vessel in clear view. The double jointed positioning rods and the sliding gantry plate can be used to provide viable placement options for the surgical microscope, while maintaining a favourable angle of approach for the micromanipulator. The camera output was streamed to a laptop, allowing the operator to alternate between a direct microscopic view and live-screen guidance. Streaming to the laptop also allowed other researchers to follow the experiment in real time. Fig 5 shows the integration of the surgical microscope and the attached camera for cannulation of sub-millimetric vessels and livestreaming of the experiment.

### 3D printing information (adaptor piece for gantry plate)

Parts were fabricated by fused-filament fabrication (FFF) on a Bambu Lab P1S printer (Bambu Lab EU, London, United Kingdom) using a 0.4 mm stainless-steel nozzle and Bambu Studio v02.03.01.51. The material was Bambu PLA Basic (Ø 1.75 mm). Slicing parameters: layer height 0.16 mm (first layer 0.20 mm); typical line width 0.42 mm (first layer 0.50 mm); 2 perimeters; 6 top and 4 bottom solid layers; infill 15% (grid). Supports were disabled; adhesion used a 5 mm brim. Temperatures: nozzle 220 °C (first layer 220 °C); bed 55 °C on Textured PEI plate. Motion settings (slicer targets): outer wall 200 mm/s, inner wall 300 mm/s, infill 330 mm/s, travel 500 mm/s; default acceleration 10 000 mm/s² (outer wall 5 000 mm/s², initial layer 500 mm/s²); seam aligned; Z-hop 0.4 mm; elephant-foot compensation 0.15 mm. The print comprised 75 layers to a maximum Z of 12.04 mm; estimated total time was 30 min 31 s, with 12.16 g of filament used. For the detailed 3D printing information of the 12–10 mm conversion piece to attach the micromanipulator to the rod with a 10 mm diameter, please see S1 file.

### Anatomical specimen information

Three Thiel embalmed human bodies were used to evaluate the set up in the neck, subclavicular and axillary region, which contain the subclavian lymphatic trunk. Thiel embalming retains joint mobility, preserves tissue plasticity and color and prevents putrefaction [25]. Thiel embalmed bodies can be preserved for several years. This allows multiple dissections, contrasting experiments and medical imaging acquisition to be performed over an extended period of time [6,7,16]. Fresh frozen specimens were considered unsuitable for these experiments, as the tissues decay too rapidly to accommodate the experimental timeline of a lymphatic mapping experiment [6]. The retained range of motion of the joints in Thiel embalmed specimens also makes it possible to reposition the body during dissection, to create additional dissection approaches. Conventional formaldehyde embalming produces rigid specimens as a result of the embalming process [26], restricting dissection approach possibilities. This makes Thiel embalming the preferred choice of body preservation for prolonged lymphatic mapping experiments [6,7,16]. The study included 2 female specimens (75 and 87 years old) and 1 male specimen (75 years old), all of Caucasian origin.

Bodies were obtained through the Ghent university body donation program, in which donors provide written informed consent for their remains to be used for medical education, training and research. Approval to use the specimens for the current study was obtained from the Ghent university hospital committee for medical ethics (ONZ-2023–0367). Experiments were conducted from 15-10-2024–15-07-2025. The three bodies that were used for this study were selected/’recruited’ from the donated bodies available during that time period. Experiments were performed in accordance with the 1964 declaration of Helsinki and all subsequent revisions and according to the institutional guidelines.

## Results

**Fig 6:**
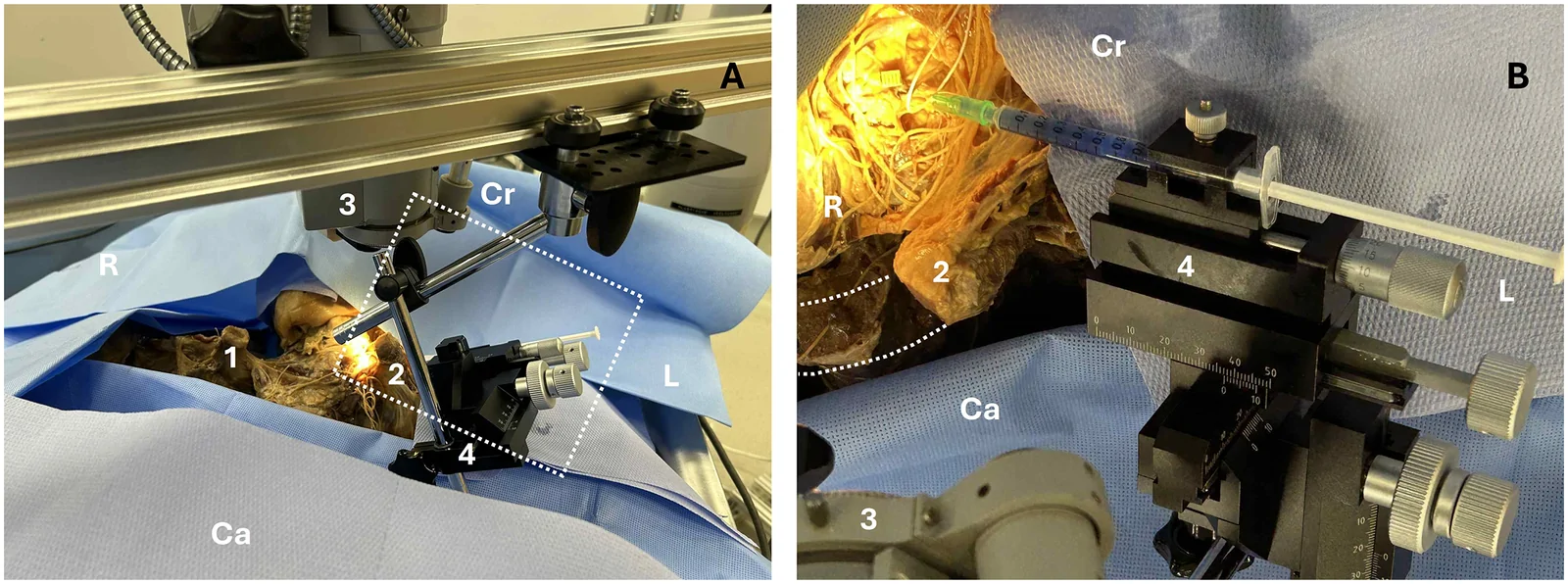
Pictures highlighting the manoeuvrability of the micromanipulator rig for the approach of a difficult to reach area for manual cannulation. Panel A shows the configuration of the rods and the micromanipulator on the rods in order to reach a space cranial to the first rib. The picture is taken from a caudolateral perspective, from the left side of the body, to show all the positioning components of the micromanipulator rig in a single picture. Cr = cranial, Ca = caudal, L = left side of the body, R = right side of the body. 1 (right side) and 2 (left side) are placed under the cutting surfaces of the posterior part of the first ribs. (The sternum and the anterior part of the first ribs connected to the sternum were removed to reach the thoracic duct and venous angle.) 3 shows the bottom part of the surgical microscope. 4 shows the micromanipulator in position to cannulate a lymphatic vessel. The white dotted box highlights the area that is shown in close up in panel B. Panel B shows a close up of the region to be cannulated, located cranial to the first rib, on the left side of the body. All components of the micromanipulator rig and the position of the body are in exactly the same configuration as they are shown in panel A. For panel B however, the picture was taken from a craniolateral perspective, from the left side of the body. This gives a clear view of the needle positioned close to the lymphatic vessels in the target region. The white dotted lines show the anterior part of the first rib that was removed along with the sternum. All other abbreviations added to panel B correspond to the description of panel A.

Following a short learning period of only several hours to familiarize the operator with the available degrees of freedom and angles of approach, successful lymphatic micro cannulations were achieved with the new set up. Fig 6 shows the micromanipulator positioned to access the subclavian lymphatic trunk in an area that is unsuitable for manual cannulation. Cannulation requires precise alignment between the cannula and the target vessel. Anatomical obstructions along the cannulation path, like the first rib, often make manual cannulation impossible because they block the hand and cannula from approaching the target vessel. In addition, manual cannulation requires a stable hand support. In the thoracic region, the curvature of the chest elevates the support surface above the target vessel, forcing the operator into a steep downward approach that cannot be aligned with the vessel. Consequently, manual cannulation in this region is often not feasible. In contrast, the micromanipulator rig provides an unobstructed, precisely aligned approach to the target vessel, demonstrating the practical advantage of its enhanced manoeuvrability.

**Fig 7:**
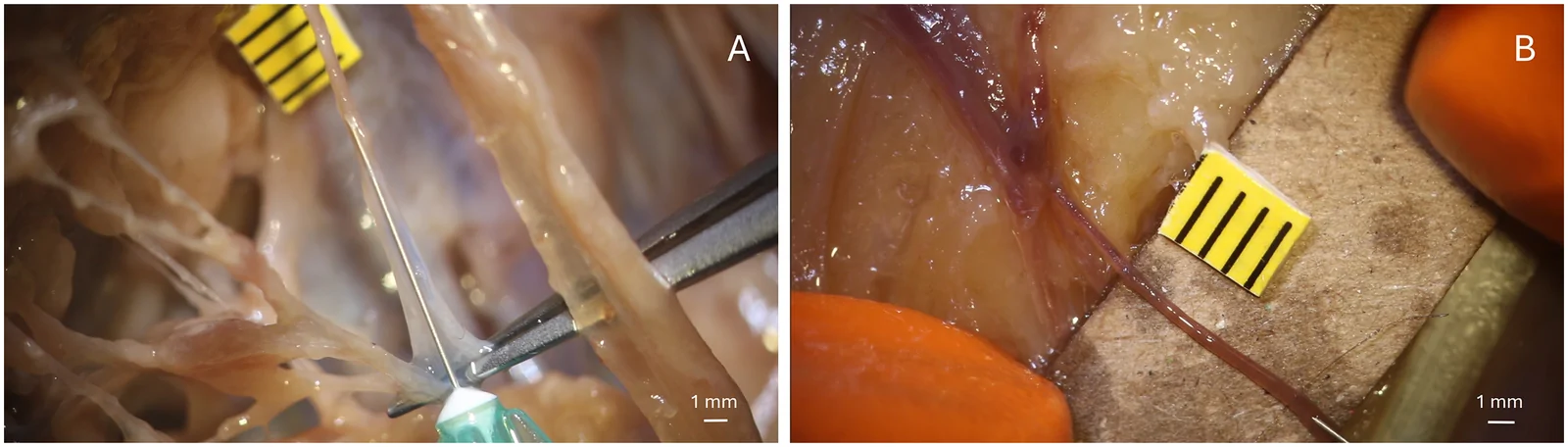
Different techniques to immobilise the target vessel during a micromanipulator rig assisted cannulation. Panel A shows immobilization of the target vessel with microsurgical forceps. This technique is useful for lymphatic vessels with a relatively short length for cannulation, due to the presence of side branches along their path. When using this technique it is easier to move the needle towards the vessel using the dials on the micromanipulator. Panel B shows immobilisation by means of positioning a rigid material underneath the vessel. The surface friction of the material keeps the vessel in place on top of it and the vessel can be cannulated by moving the rigid material towards the needle.

Apart from precise needle control, successful cannulation also required adequate stabilization of the target vessel. Without stabilization, the needle displaced the vessel instead of penetrating the vessel wall. As such, the vessel had to be aligned with the needle and placed under slight tension during cannulation. Two effective stabilization techniques were identified (Fig 7). The first method involved immobilising the target vessel and applying a slight tension in the direction of the needle, using microsurgical forceps (Fig 7A). For this approach the needle was moved into the aligned, immobilized vessel, by using the dials on the micromanipulator. This approach was suitable for shorter vessels, or vessels with several side branches. The second method uses the addition of a small piece of rigid material under the vessel. For the second method, the vessel is moved towards the needle by moving the supporting material with the vessel on top of it, instead of operating the micromanipulator. Because the vessel is immobilized by the friction of the supporting material’s surface, the vessel wall can be punctured by pulling the vessel against the needle, which is kept perfectly stationary by the micromanipulator (Fig 7B). This technique proved more suitable in cases where a longer lymphatic vessel, without side branches needed to be cannulated.

**Fig 8:**
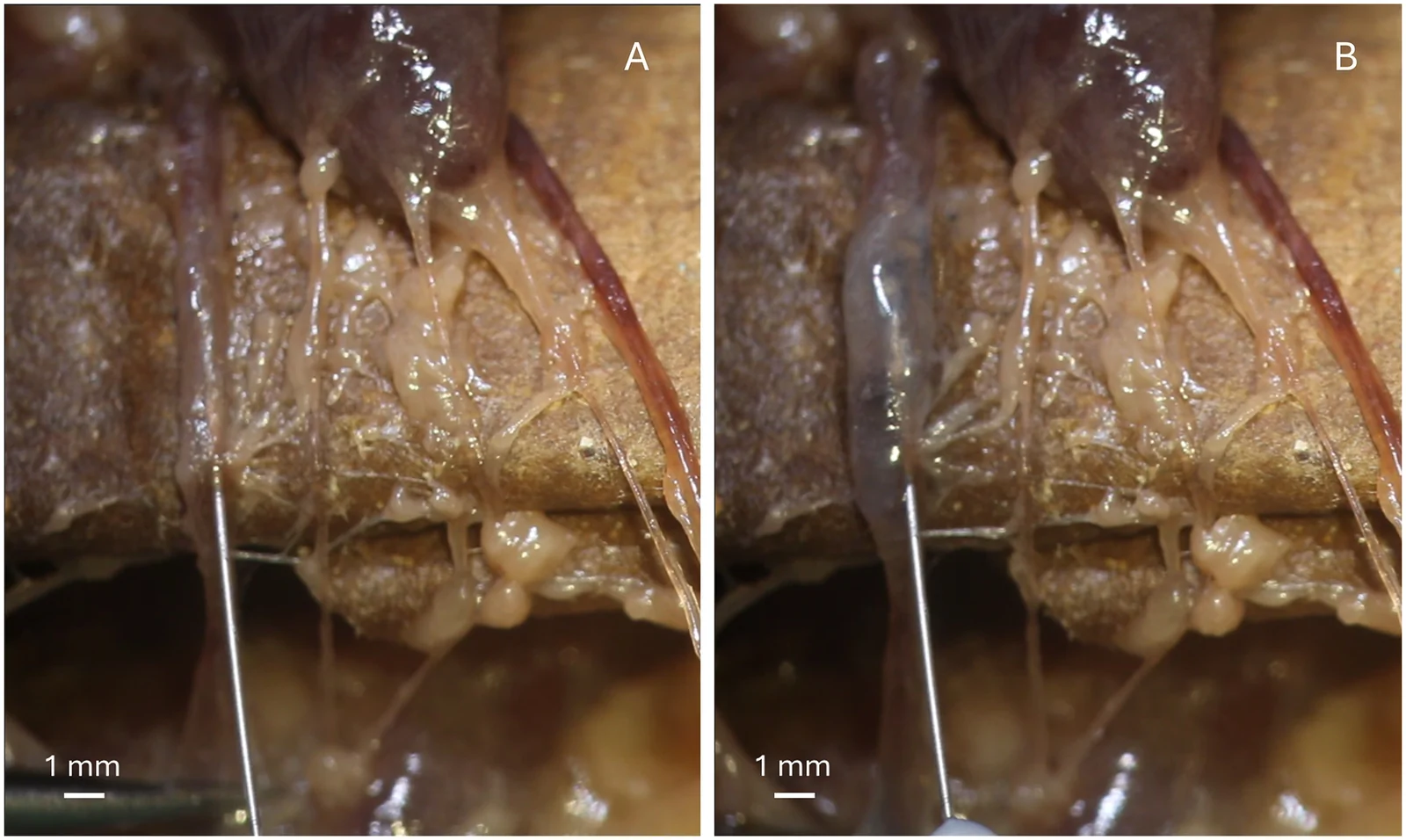
Cannulation of a lymphatic vessel with rigid material under the vessel, during a contrast agent injection. Panel A shows a successfully cannulated lymphatic vessel before contrast agent injection. Panel B shows the same vessel during injection of contrast agent, with the lymphatic vessel wall distended to double the original size of the vessel.

Finally, Fig 8 shows a cannulated lymphatic vessel, before and during contrast agent injection using a 33G needle. Fig 8 gives a good example of the expansion that the lymphatic vessel wall can display. This highlights the importance of pressure control during contrast agent injection, because the delicate vessel wall can easily rupture if the contrast agent is added using excessive/uncontrolled injection pressure [7,16]. When controlled injection pressure is required, the cannulation set up shown in Fig 3A, can be replaced with the cannulation set up in Fig 3B, connected to a pressure control device.

Most importantly, all canulations in the neck, subclavicular and axillary region, could successfully be completed using the micromanipulator rig set up on a full body specimen. Obstructions of the cannulation path, or a difficult path of approach in the axillary region make most manual cannulations unfeasible. By leveraging the manoeuvrability of the micromanipulator rig set up, these obstacles could successfully be avoided. In general, due to the added stability provided by the micromanipulator and the manoeuvrability of the micromanipulator rig set up, cannulations were easier to perform and quickly achieved a higher success rate than manual cannulations in the same region.

## Discussion

Several regions of the human lymphatic system have remained anatomically understudied for centuries, highlighting the need for updated anatomical descriptions/depictions [2,5]. This knowledge gap is likely related to the available methodologies for contrast agent injection. The current methodologies are restricted to body regions that can be positioned under a benchtop microscope and that are easily accessible on the surface of the specimen. Most lymphatic anatomical data is provided for superficial lymphatics in the extremities [9,27,28], or on the thoracic duct that can more easily be cannulated manually, due to its comparatively large size [6,15–17]. Deep lymphatic structures like the subclavian lymphatic trunk remain understudied, despite its clinical relevance for breast cancer treatment [7]. The SLT runs deep within the tissues, with the first rib, the clavicle and the curvature of the ribs obstructing the cannulation path. It is also present in a region of the body that cannot be positioned under a benchtop microscope. As a result, cannulation has relied on manual techniques that lack the accuracy/stability required for sub-millimetric vessels and frequently fail [24]. Failed cannulations not only waste hours of meticulous micro dissection, but also render specimens unsuitable for contrast enhanced imaging and subsequent 3D reconstruction, due to contrast leakage.

To address these limitations, we developed a full body micro cannulation set up, using a multidisciplinary approach. The set up was validated in the neck, subclavicular and axillary region. To the best of our knowledge this is the first set up to perform full body micro cannulations in a human anatomy lab. By combining a suspension rig with a sliding gantry plate that holds the micromanipulator above the full body test set up, all the required degrees of freedom to provide successful micro cannulations were achieved. The stable frame, combined with the micromanipulator prevents tremors during needle/cannulation movement, providing a dependable new set up for micro cannulation in a full body setting. Compatibility with different micro cannulation equipment (Fig 3), options to include a surgical microscope and a pressure control device make the set up very versatile and allow easy cannulation of different sizes of sub-millimetric vessels. The degrees of freedom provided by the overhead design allow enough angles of approach to avoid obstructed cannulation paths that would have hindered manual cannulations. The set up is easy to use and more accurate than manual micro cannulation. After only several hours of practice, the success rate of micro cannulations using the micromanipulator rig surpassed that of manual micro cannulations in the same region, greatly facilitating the contrast agent injection part of lymphatic mapping experiments. The micromanipulator rig will therefore be a valuable tool for future lymphatic mapping studies in regions that were previously underexplored.

Only one similar set up for micro cannulations of human lymphatic vessels was described in the literature, but this set up was not for use on full bodies. Instead, it was focused on superficial lymphatic vessels and its use was intended for sectioned (extremity) specimens, or even smaller specimens [24]. The micromanipulator rig can be used for small lymphatic vessels across the entire body, but it is also possible to use it on smaller separate sections of a body. Apart from using the micromanipulator rig for lymphatic vessels, it can also be used for venules and arterioles if there is a need for this. The micromanipulator rig enables the reliable introduction of contrast agent into sub-millimetric vessels. Its functionality is complimented by the option to include a pressure control device and the option to take microscopic photos/videos during experiments. These additional options enable the study of contrast agent propagation inside the lymphatic vessels and could provide additional insight into sub-millimetric (lymphatic) vessel and valve properties in an in situ, human cadaver setting. These capabilities should make the micromanipulator rig an asset for every anatomy department with the need for a reliable and versatile alternative for micro cannulations.

The goal of designing the micromanipulator rig was to facilitate micro cannulations in an anatomical research lab and to be functional and easy to use for this specific purpose. For this reason it was built with as little different components as possible, to facilitate assembly and ease of operation. We are aware that operating rooms have access to more sophisticated systems with similar functionality, like surgical robots. A surgical robot can likely reach the same degrees of freedom and angles of approach for micro cannulation, but integrating it in an anatomy lab for basic anatomical research is unrealistic, so this was not considered.

Because of its superior accuracy and manoeuvrability, compared to manual cannulations and its ease of use, the micro manipulator rig will be very useful for future lymphatic mapping studies. It will allow previously understudied regions of the lymphatic system to efficiently be filled with contrast agent. This will enable MRI + (micro)CT scans that can be used to create accurate 3D depictions of the lymphatics in relation to the surrounding anatomy [23]. This will in turn provide more accurate depictions of the lymphatic system that can help radiotherapists and oncologic surgeons to devise better/more accurate cancer treatments.

## Conclusion

A micromanipulator rig consisting of a suspension rig, a gantry plate, positioning rods and a micromanipulator was created for micro cannulations in a full body setting. All attempted cannulations in the neck, subclavicular and axillary region were possible due to the manoeuvrability and stability of the micromanipulator rig. The set up was also easy to use. Micro cannulations were possible in a full body setting, even in regions that are very difficult to cannulate manually, due to the obstruction of the cannulation path. Following a short learning period, a successful cannulation was much easier to achieve compared to a manual cannulation in the same area. The option to add a surgical microscope and a pressure control device, combined with compatibility for different cannulation equipment make the set up very versatile. Vessels of different sizes can be cannulated, experiments can be recorded and livestreamed and contrast agent propagation studies can be performed to obtain in situ experimental data on (lymphatic) vessels and valves from human cadaveric specimens. We would therefore recommend using the micromanipulator rig set up for future microvascular experiments and especially for future lymphatic mapping studies.

## Supporting information

S1 File 3D printer settings for printing the conversion piece to fit the micromanipulator with a 12 mm diameter opening onto the positioning rod with a 10 mm opening. (DOCX)
