# Supplementary material for: Construction of an experimental set up to perform full body micro cannulations of sub-millimetric vessels in an anatomical research setting
Source: PLoS One. 2026 Sep 11;21(9):e0358264. doi: 10.1371/journal.pone.0358264 (PMC13567826; doi:10.1371/journal.pone.0358264)
Supplement: Supplementary file 1 [file pone.0358264.s001.docx]

**3D printing parameters for 12 to 10 mm adapter piece.**

- Printer & slicer: Bambu Lab P1S; Bambu Studio v02.03.01.51

- Material: PLA (Bambu Lab profile); filament Ø 1.75 mm; density 1.26 g/cm³ (profile)

- Nozzle & extruder: 0.4 mm stainless-steel nozzle; Direct Drive extruder

- Layer height: 0.08 mm; first layer 0.20 mm

- Line widths: typical 0.42 mm; outer wall 0.42 mm; inner wall 0.45 mm; first layer 0.50 mm

- Shells: bottom 7 layers (top layers not specified in this file)

- Infill: internal solid zig-zag pattern; infill direction 45°; wall overlap 15%

- Supports: Disabled

- Temperatures: Nozzle 220 °C (including first layer); Bed 55 °C; build plate Textured PEI Plate

- Speeds: outer wall 200 mm/s; inner wall 350 mm/s; internal solid infill 350 mm/s; initial layer 50 mm/s; bridge 50 mm/s; overhang speeds 60/30/10/10 mm/s; ironing 30 mm/s

- Acceleration & jerk: default accel 10,000 mm/s²; outer wall 5,000 mm/s²; initial layer 500 mm/s²; initial travel accel 6,000 mm/s²; machine jerk X/Y/Z/E = 9/9/3/2.5; wall and initial-layer jerk 9

- Adhesion & seam: brim 5 mm (auto-brim); apply scarf seam on circles = enabled; elephant-foot compensation 0.15 mm

- Part height: 17.32 mm max Z

- Job estimates: 21 m 35 s total (model time 15 m 13 s); 215 layers; filament length 296.04 mm; mass 0.90 g
