# Supplementary material for: Construction of an experimental set up to perform full body micro cannulations of sub-millimetric vessels in an anatomical research setting
Source: PLoS One. 2026 Sep 11;21(9):e0358264. doi: 10.1371/journal.pone.0358264 (PMC13567826; doi:10.1371/journal.pone.0358264)
Supplement: Supplementary file 2 [file pone.0358264.s002.docx]

**Response to reviewers PONE-D-25-67491 R1**

The authors wish to thank the editor and the reviewers for their thorough review and their comments for the improvement of the manuscript. Responses to each specific question/remark are positioned under the question/remark, preceded by •.

*Editor comments*

When submitting your revision, we need you to address these additional requirements.

1. Please ensure that your manuscript meets PLOS ONE's style requirements, including those for file naming. The PLOS ONE style templates can be found at

<https://journals.plos.org/plosone/s/file?id=wjVg/PLOSOne_formatting_sample_main_body.pdf> and 
<https://journals.plos.org/plosone/s/file?id=ba62/PLOSOne_formatting_sample_title_authors_affiliations.pdf>

- We have adhered to all style requirements listed in the links above.

2. Thank you for stating the following financial disclosure:

“MEJS is funded by Research Foundation Flanders, grant number 1202224N.”

Please state what role the funders took in the study.  If the funders had no role, please state: "The funders had no role in study design, data collection and analysis, decision to publish, or preparation of the manuscript." If this statement is not correct you must amend it as needed.

- The financial disclosure statement: “MEJS is funded by Research Foundation Flanders, grant number 1202224N. The funders had no role in study design, data collection and analysis, decision to publish, or preparation of the manuscript.”

Please include this amended Role of Funder statement in your cover letter; we will change the online submission form on your behalf.

- This was added to the cover letter.

3. We note that Figures 2 to 8 in your submission contain copyrighted images. All PLOS content is published under the Creative Commons Attribution License (CC BY 4.0), which means that the manuscript, images, and Supporting Information files will be freely available online, and any third party is permitted to access, download, copy, distribute, and use these materials in any way, even commercially, with proper attribution. For more information, see our copyright guidelines: <http://journals.plos.org/plosone/s/licenses-and-copyright>.

- This copyright statement was only applicable to figure 2, where figures from the supplier’s online catalog were used in some panels (to pinpoint exactly which components were used). Thank you for pointing this out, as I did not initially take this into account. Figures 3-8 are based on photographs that were taken by the author himself, of (parts of) the set up that was constructed by the authors and described in the paper. As such the authors already own the copyright to these figures.
- The panels in figure 2 that contained figures from the supplier’s website were replaced with photographs taken from the separate components. Photographs were taken by the author, to ensure that the copyright for all figures in the manuscript now completely belongs to the authors. As such, the suggested written permissions of the copyright holders are no longer required.

4. Please include captions for your Supporting Information files at the end of your manuscript, and update any in-text citations to match accordingly. Please see our Supporting Information guidelines for more information: <http://journals.plos.org/plosone/s/supporting-information>.

- Captions for the supplementary files were added to the end of the manuscript, below the references.

5. We are unable to open your Supporting Information file [Gantry Plate Adapter CAD model.STL, 12-10adapter CAD model.STL]. Please kindly revise as necessary and re-upload.

- The STL files also caused problems during the technical check, upon resubmitting the revised version of the manuscript. This is understandable, because this is a very technical 3D file format that requires specific software to open it.
- It was therefore decided to leave the STL files out of the supplementary material of the revised submission, to prevent technical issues. If other researchers want to print the 3D pieces, they will have this specific software available and they can request the files from the corresponding author. We will happily oblige these requests.

6. If the reviewer comments include a recommendation to cite specific previously published works, please review and evaluate these publications to determine whether they are relevant and should be cited. There is no requirement to cite these works unless the editor has indicated otherwise.

- No additional references were requested/recommended, so this was not applicable.

**Additional Editor Comments:**

All reviewers found merit in the study, but reviewer 1 is concerned about the real-case scenario applicability of the setup. I would suggest that authors elaborate this aspect of their work, as detailed at the reviewer's suggestions below.

- Please see the response to the specific comments of the reviewers below.

Reviewer 1

Reviewer #1: Authors describe a microcannulation rig composed of a mechanical micromanipulator attached to a rigid frame capable of greater degree of motion to reach and successfully inject liquids in small, poorly accessible lymphatic vessels. Albeit their work shows a very interesting and meaningful proof of concept, several major questions arise from the paper which need to be addressed to help readers clearly appreciate why the proposed solution is to be preferred over others.

Another major point is that it is never mentioned how to operate the device in sterility, since Authors performed the proof on cadavers and, from the images presented in the manuscript, no sterile environment has been set up. Author should discuss sterility of the micro manipulator, the gantry system, the 3D printed part. Moreover, how would the rig perform when the subject is equipped with monitoring devices and their associated connections in a real surgical setting? All these aspects must be taken into account so that readers can judge the actual feasibility of operating the system in a real environment. Another major point is the suggested use of pulled glass micropipettes (line 176). Extremely fine glass tips may and will eventually break, and should this happen while cannulation of the lymphatic vessel is in progress, how this complies with safety requirements during real surgery? Are there alternatives to glass pipettes, apart from needles? Authors must address this point if they propose the use of this system in a real surgical setting.

- The set up was never intended to be used in the operating room/a surgical setting. It is specifically meant to be used in a gross anatomical research setting in the anatomy lab, in order to help generate an anatomically accurate 3D representation of the lymphatic system. This means that there is no need to ensure sterility, comply with OR conditions, or comply with safety regulations regarding the components (glass needles) of the set up.

Lymphatic data acquired with the set up can at a later stage be used to inform lymphatic interventions/cancer treatment. However, these lymphatic data are solely intended to better depict lymphatic anatomy in relation to the surrounding anatomy in 3D reconstructions based on the cadaveric specimens. A cadaveric set up is better suited for this specific purpose, as it allows a broader range of contrast agents (substances that would be harmful to a patient) that can give a much more clear/brighter signal than clinical contrast agents in the safe dose-range for a patient. Anatomical specimens also allow a wider variety of dissection approaches to the lymphatic vessels than the approaches that would be impossible in patients (like partially removing the clavicle for better access). Unlike patients, the anatomical specimens can be CT scanned without taking the radiation doses into account. Scanning cadaveric specimens injected with (non-clinical) contrast agent using the proposed set up will therefore yield higher quality CT scan data due to (the possibility of) longer scan times and the additional absence of movement artefacts.

- Due to their small size, lymphatic vessels are not visible on medical imaging (CT/MRI) and a 3D visualization cannot be reconstructed from the scan data if the lymphatic vessels do not contain contrast agent. This makes the ability to inject contrast agent into the lymphatic system the most vital part of the research trajectory to create a 3D model of the lymphatic system in relation to the surrounding anatomy. As we encountered practical difficulties during previous manual cannulation attempts of sub-millimetric lymphatic vessels (both in relation to wrist/hand tremor and in angles of approach for cannulation of these vessels) it was clear that a solution needed to be created for this purpose.

The set up was specifically developed to assist in micro-cannulation of sub-millimetric lymphatic vessels on full body cadaveric specimens in the anatomy lab.

- To the best of our knowledge glass needles are the only devices for cannulation capable of attaining a diameter below that of a 33 gauge needle (smallest commercially available needle). Even though they are fragile, they are suitable for use in a cadaveric set up, where there is no longer any safety concern for the specimen.

First of all, it is not clear wether the main point is to have a very versatile system or to limit the costs associated with such a device, given that surgical robotic arms are already present and can perform the same tasks with all the safety requirements in order to be operated in a surgical room. Moreover, Authors do not consider alternatives to the mechanical manipulator that they propose. It would be beneficial if a discussion on why servo-actuated micro manipulators controlled by joystick or other 3D control devices operated at some distance from the site of the cannulation could be incorporated in the manuscript, to orient the reader. Also, no discussion on alternative geometries of the suspension rig have been proposed, and again this point has to be faced to let the reader understand why Authors chose the linear rig. (This remark was moved below the previous remark to better structure the answers to your questions)

- The purpose of creating the set up was not to improve on existing systems/replace existing systems, or to create a cheaper version of existing systems. It was to create a set up that would be useful for the specific purpose we intended it for: reliably cannulating sub-millimetric lymphatic vessels with enough angles of approach for a full body cadaveric setting in the anatomy lab. We did not compare it to other alternatives, as it was able to perform the actions we needed it to and it allowed us to continue our research. The main criteria that were used for the creation of the set up were functionality and practical use, so the design was kept as simple/practical as possible with as little separate parts as possible. This approach led to the linear suspension rig design with the sliding gantry plate.
- Including servo activated components could perhaps improve the set up, but it would introduce several possible points of failure/complexity and it could delay the construction of the set up and the continuation of the research project by months. In other words: once the set up did what we needed it to do, no other adaptations were needed and it was used to continue the lymphatic injections for our research project.
- Even though a surgical robot could perhaps achieve the same outcomes as our set up, equipping an anatomy department (per definition a non-sterile environment) with a very expensive surgical robot for the purpose of basic lymphatic research does not seem realistic. When a surgical robot is available, it would very likely be more beneficial to use it in a surgical setting for the direct benefit of patients.
- A paragraph was added to the discussion to explain that the current set up was created specifically for use in an anatomical research lab and that using a surgical microscope for basic anatomical research would not be feasible in this setting.

Methods are not properly written, a more specific and punctual style could have been adopted.

- The materials and methods were rewritten and additional sub headings were added to achieve a more specific and punctual style.

English has to be revised for fluency.

- English was revised for fluency and adapted where necessary.

Overall, in this Reviewer's mind, Authors propose a very interesting proof of concept, but the work and the manuscript are still at a too early stage of development to be of significant impact in a real scenario.

- The work is at the stage where it was useful for its intended purpose and the goal of the manuscript was to spread it to the anatomical community. Anatomists can benefit from the set up if they are doing similar basic anatomical research and have encountered a similar problem with the injection of small vessels. The real scenario here is the adoption of the set up in an anatomy lab.

*Reviewer #2:*

1. Grammar and language should be checked once by authors. I have given an example {An unobstructed approach for this normally very difficult position for cannulation} you can write either very difficult or more difficult.

- English was revised for fluency and adapted where required.

2. please edit and make introduction catchy and short. you can add more details in conclusion and discussion section

- The introduction was rewritten to shorten it by half a page and make it more catchy.

*Reviewer #3*

This paper deals with an original methodological issue, with the proposal of a new set up for lymphatic vessel micro cannulations. The manuscript is well organized and written clearly enough to be accessible to non-specialists. Anyway, some minor changes are needed to improve the paper.

This article provides a new method for micro cannulating lymphatic vessels. Historically, the perfusion of lymphatic vessels has always presented a major technical challenge. This work advances the current knowledge on this topic. The authors suggest the use of an innovative tool for microvascular researchers and have a robust experience on this issue. The new method is described in detail with several pictures of high quality. The authors conclude that their new tool represents an improvement with respect to previous approaches. This conclusion is supported by an accurate technical and functional description of the tool. References are appropriate and accurate.
Any additional comments.

Caption of figure 6 is not clear to the reader. Which side (left, right) of the body is represented? Once specified the side, use the anatomical terms medial and lateral to indicate the position of a structure. In panel A, part of the first rib was removed and 1 and 5 are its lateral (vertebral) and medial (sternal) stumps. Right?

- Figure 6 was reworked and the figure legend was adapted to improve clarity.

Panel A of figure 7: 1 mm instead of 1mm, as in panel B. Is yellow paper a scale?

- Panel A was adapted as requested. Yes, the yellow paper is a scale. The space between 2 adjacent black lines equals 1 mm. This scale was used to create the scalebar on the bottom right side of the picture for additional clarity.

Did the author patent their device?

- No, the device was not patented as the authors hope it can help other anatomical researchers with their research. Especially since the field of anatomical lymphatic research is severely understudied at the moment. This likely has to do with the accessibility of the sub-millimetric lymphatic vessels, which our set up remedies. This is also why the materials and methods section was written in high detail, to allow the device to be replicated by the readers.

The special characteristics of Thiel embalmed bodies are unfamiliar to most readers. The authors are invited to provide more information.

- Additional information about the Thiel embalming technique was added to the materials and methods section.

Additional information about the three embalmed bodies must be provided: age, gender, ethnic group, etc.

- Information about gender, age and ethnicity of the specimens was added.
