# Supplementary material for: Construction of an experimental set up to perform full body micro cannulations of sub-millimetric vessels in an anatomical research setting
Source: PLoS One. 2026 Sep 11;21(9):e0358264. doi: 10.1371/journal.pone.0358264 (PMC13567826; doi:10.1371/journal.pone.0358264)
Supplement: Supplementary file 3 [file pone.0358264.s003.docx]

**Response to reviewers PONE-D-25-67491 R2**

The authors wish to thank the editor and the reviewers for their thorough review and their comments for the improvement of the manuscript. Responses to each specific question/remark are positioned under the question/remark, preceded by •.

Reviewer #1: Authors have cleared the intended scope of their setup; their responses have fulfilled all the relevant issues that have arisen from the previous version of the manuscript. Just one remaining point might be modified, and it is the reference to the use of the present setup to study lymph flow: usually, this task is performed in “in vivo” or “ex vivo” experiments, where lymph flow is properly driven by intrinsic and extrinsic pumping mechanisms. Referring to flow in this context could be misleading since the only flow that could be appreciated would be the one induced by the injection of liquids into the cannulated vessel, thus not a physiological one. If the intended meaning was to experimentally induce flow inside the cannulated lymphatic vessels to study the anatomical path of the regional lymphatic network, this could be better defined in the title and text without the reference to flow.

- All mentions of lymphatic flow were replaced with more appropriate terminology (intralymphatic contrast agent propagation) for clarity. The title was also rewritten, removing the mention of lymphatic flow studies and highlighting the intended use in an anatomical research setting.

Reviewer #3: The authors provided a careful revision of their manuscript. Changes have been highlighted in the text. The anatomical terminology is now satisfactory.

- Thank you.
